# RNA-seq Analysis of Wild-Type vs. FOXC2-Deficient Melanoma Cells Reveals a Role for the FOXC2 Transcription Factor in the Regulation of Multiple Oncogenic Pathways

**DOI:** 10.3389/fonc.2020.00267

**Published:** 2020-02-27

**Authors:** Kristian M. Hargadon, Corey J. Williams

**Affiliations:** Hargadon Laboratory, Department of Biology, Hampden-Sydney College, Hampden-Sydney, VA, United States

**Keywords:** RNA-seq, melanoma, FOXC2, gene regulation, oncogene, differential expression

## Introduction

The forkhead box protein C2 (FOXC2) transcription factor has recently emerged as a key regulator of tumor progression in many cancer types. First implicated as a potential oncogenic transcription factor due to its overexpression/nuclear localization in invasive breast carcinomas, particularly those of the aggressive basal-like subtype (1), FOXC2 has since been linked to the progression of a number of epithelial-derived malignancies. Indeed, FOXC2 overexpression and nuclear localization are poor prognostic indicators of survival in patients with prostate cancer (2), hepatocellular carcinoma (3), NSCLC (4), colorectal cancer (5), glioma (6), gastric cancer (7), and esophageal as well as oral tongue squamous cell carcinomas (8, 9). Studies employing murine and human tumor cell lines have confirmed the oncogenic potential of the FOXC2 transcription factor, highlighting its ability to promote several hallmarks of cancer progression, including proliferation (5, 9), epithelial-mesenchymal transition (EMT) (10), invasion and metastasis (11), glycolytic metabolism (12), stemness (13), and drug resistance (14, 15). Based on these diverse tumor-promoting functions and the breadth of tumor types in which FOXC2 is dysregulated, it is important to improve our understanding of this transcription factor's regulation of oncogenic pathways in cancer cells.

While previous studies have focused on FOXC2 in the context of cancers originating from epithelial tissues, we recently demonstrated that FOXC2 is also a key contributor to the progression of melanoma (16). Using CRISPR-Cas9 gene editing technology, we engineered a variant of the B16-F1 murine melanoma cell line that carries a bi-allelic disruption in the *Foxc2* gene and that does not express FOXC2 protein, and we reported that this B16-F1ΔFOXC2 variant grows out with slower kinetics as a subcutaneous tumor than its parental counterpart. We also reported select data from RNA-sequencing (RNA-seq) and pathway-focused qRT-PCR array-based differential gene expression studies in the B16-F1 vs. B16-F1ΔFOXC2 melanomas that highlighted a role for FOXC2 in: (1) the positive regulation of genes associated with the cellular response to xenobiotics and oxidative stress and (2) the negative regulation of genes associated with interferon (IFN) responsiveness. These findings were particularly intriguing in light of our analysis of data from The Cancer Genome Atlas (TCGA), which revealed that *FOXC2* expression levels correlate negatively with survival of melanoma patients treated with either dacarbazine chemotherapy or ipilimumab immunotherapy. In this Data Report, we now provide a more thorough description of our RNA-seq data obtained from the B16-F1 and B16-F1ΔFOXC2 melanoma cell lines. Importantly, these data reveal a role for FOXC2 in the regulation of multiple pathways with oncogenic potential in melanoma, and they offer mechanistic insights into FOXC2-associated tumor progression that may be applicable to other cancer types as well.

## Methods

### Cell Lines

B16-F1 murine melanoma cells were purchased from the American Type Culture Collection (Manassas, VA, USA) and grown in RPMI-1640 medium supplemented with 2 mM L-glutamine, 2 g/l glucose, and 2 g/l sodium bicarbonate (Thermo Fisher Scientific, Waltham, MA, USA), as well as 10% fetal bovine serum (Premium Select, Atlanta Biologicals, Norcross, GA, USA). B16-F1ΔFOXC2 cells were generated as described (16) and maintained in the same growth medium as the parental cell line. All cultures were grown at 37°C in a 5% CO_2_ incubator and passaged at 80–90% confluence.

### RNA Isolation

B16-F1 or B16-F1ΔFOXC2 melanoma cells (1e^6^) were plated onto 60 × 15 mm cell culture dishes and grown for 24 h to ~90% confluence before isolating RNA with an RNeasy Mini Kit (Qiagen, Germantown, MD, USA) according to the manufacturer's recommendations. On-column DNase-digestion with Qiagen's RNase-free DNase Set was performed during extraction. RNA integrity and genomic DNA contamination were examined by standard denaturing agarose gel electrophoresis, and all samples (five independent replicates per group) passed quality control assessment. RNA was quantified with an Epoch Spectrophotometer (BioTek, Winooski, VT, USA), and A260/280 and A260/230 ratios were both ≥2.0 for all samples.

### Preparation of Libraries for RNA-seq

RNA samples were shipped overnight on dry ice to Arraystar, Inc. (Rockville, MD, USA) for analysis using the company's Illumina Hi-seq 6G RNA-sequencing service. mRNA was isolated from total RNA (1–2 μg per sample) with oligo (dT) magnetic beads using the NEBNext® Poly(A) mRNA Magnetic Isolation Module (New England BioLabs, Ipswich, MA). RNA was fragmented to sizes between 400 and 600 bp and reverse transcribed into 1st strand cDNA using random hexamer primers according to manufacturer recommendations in the KAPA Stranded RNA-Seq Library Prep Kit (Illumina, San Diego, CA). Using this kit, 2nd strand synthesis was performed to incorporate dUTP into strand-specific libraries, and the double-stranded cDNA was end-repaired, A-tailed, adaptor ligated, and PCR amplified. Completed libraries were qualified with an Agilent 2100 Bioanalyzer using the Agilent DNA 1000 Kit (Agilent, Santa Clara, CA) and quantified by absolute quantification qPCR. Barcoded libraries were mixed in equal amounts, denatured to single stranded DNA with 0.1 M NaOH, loaded onto channels of the flow cell at 8 pM concentration, and amplified *in situ* using a TruSeq SR Cluster Kit v3-cBot-HS (Illumina). Sequencing was carried out by running 150 cycles for both ends on an Illumina HiSeq 4000 instrument.

### RNA-seq Data Processing and Analysis

Image analysis and base calling were performed using Solexa pipeline v1.8 (Off-Line Base Caller software, v1.8). Sequence quality was examined using FastQC software (v0.11.7), and raw sequencing data that passed Illumina chastity filtering were analyzed. Fragments were 5′, 3′-adaptor trimmed and filtered ≤ 20 bp reads with cutadapt software (v1.17). The trimmed reads were mapped to reference genome GRCm38 using Hisat 2 software (v2.1.0). Transcript abundances for each sample were estimated with StringTie (v1.3.3), and the normalized expression level (FPKM value) of known genes was calculated with the R package ballgown (v2.10.0). An FPKM mean of ≥0.5 in a given biological group was used to calculate the number of identified genes per group. Using these identified genes, differential gene expression analysis was performed with ballgown and the following cutoffs to filter differentially expressed genes: fold change ≥ 1.5, *p* ≤ 0.05, and mean FPKM ≥ 0.5 in at least one group. Gene ontology (GO) enrichment analysis of differentially expressed genes was performed using standard GO Terms from the Gene Ontology Resource (http://www.geneontology.org) and a Fisher's exact test to estimate statistical significance of the enrichment of terms between the B16-F1 and B16-F1ΔFOXC2 cell lines. Similarly, pathway analysis of differentially expressed genes was performed using the Kyoto Encyclopedia of Genes and Genomes (KEGG) database, and a Fisher's exact test was used to estimate the statistical significance of pathways enriched with differentially expressed mRNAs between the two cell lines.

### Data Deposition

RNA-seq data discussed in this publication have been deposited in NCBI's Gene Expression Omnibus (17) under Dataset Name “RNA-seq Analysis of Differential Gene Expression in Wild-type Versus FOXC2-deficient B16-F1 Melanomas” and are freely accessible through GEO Series accession number GSE134296, available at https://www.ncbi.nlm.nih.gov/geo/query/acc.cgi?acc=GSE134296 (18). This dataset includes both raw data in .fastq format as well as a matrix table of processed data (.xlsx format) with the normalized FPKM expression values for known genes from each sample.

## Overview and Reuse of Data

**Figure 1:**
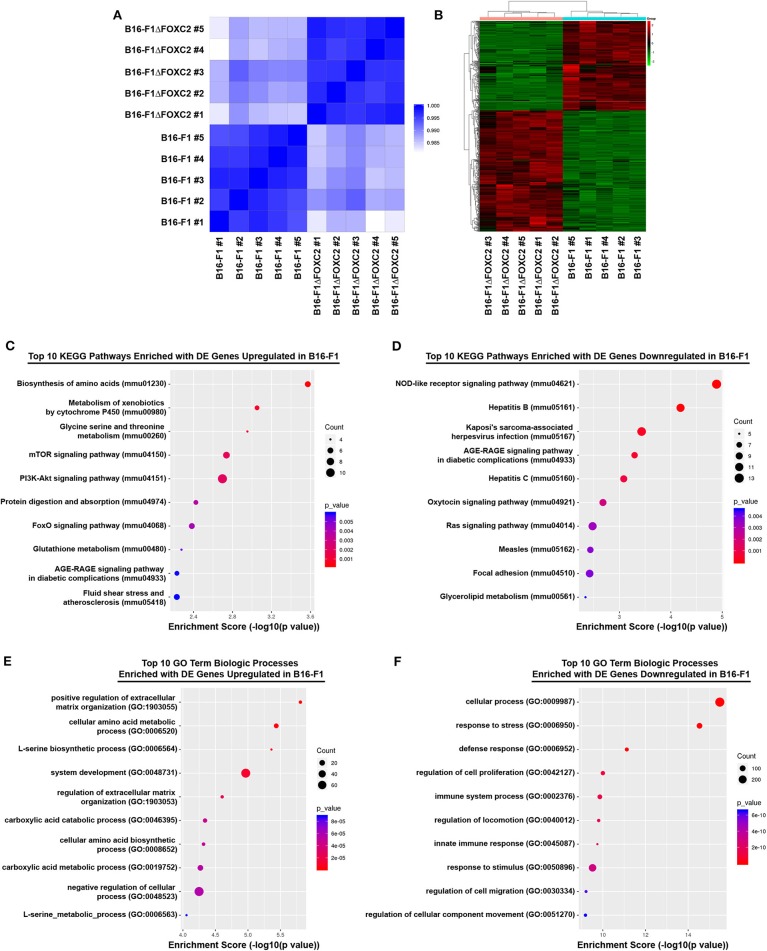
RNA-seq correlation and differential gene expression analyses of B16-F1 and B16-F1ΔFOXC2 murine melanomas. RNA-seq analysis was performed on RNA isolated from five replicate samples for each biological group. The Pearson R2 correlation heat map of gene expression levels between all samples is shown in **(A)**. The hierarchical clustering heat map of differentially expressed genes between B16-F1 and B16-F1ΔFOXC2 is shown in **(B)**. KEGG Pathway and Gene Ontology analyses were performed to identify pathways and biological processes significantly enriched with differentially upregulated and downregulated genes in B16-F1. Enrichment score dot plots showing gene counts and statistical significance as determined by a Fisher's exact test are presented for the top 10 KEGG pathways enriched in differentially expressed (DE) genes in **(C,D)** and for the top 10 Biologic Process-related GO Terms enriched in DE genes in **(E,F)**.

We recently reported that expression of the *FOXC2* gene in melanoma biopsies is an unfavorable prognostic indicator of patient survival following treatment with either chemotherapy or immunotherapy (16). In that study, we also described a novel CRISPR-Cas9 gene-edited variant of the murine B16-F1 melanoma that we engineered to lack the FOXC2 transcription factor (B16-F1ΔFOXC2). Using this model, we demonstrated a role for FOXC2 in promoting melanoma progression, and we highlighted select data from an RNA-seq analysis of the B16-F1 and B16-F1ΔFOXC2 melanomas that we now describe here in more detail. With 5 replicate RNA samples isolated from each tumor cell line, a Quality score of Q30 >82% for each sample (Q30 = 99.9% base calling accuracy), and a high level of correlation between samples within each biological group (Pearson R2 correlation > 0.993 between replicates, Figure 1A), this dataset provides a high-quality profile of the FOXC2-associated transcriptome in melanoma cells, and it will serve as a useful tool to investigators interested in studying FOXC2 function in the context of cancer.

In the differential gene expression analysis of our RNA-seq data, we defined B16-F1ΔFOXC2 as the reference sample so that genes upregulated in the wild-type B16-F1 cell line could be interpreted as those positively regulated (directly or indirectly) by FOXC2, whereas genes downregulated in B16-F1 would represent those negatively regulated by FOXC2. We identified 598 genes differentially expressed (fold-change ≥ 1.5, *p* ≤ 0.05, and mean FPKM ≥ 0.5 in at least one group) by these cell lines: of these, 254 genes were upregulated in B16-F1, implicating a role for FOXC2 in their induction, and 344 genes were downregulated in B16-F1, reflecting FOXC2-associated repression of these genes (Figure 1B). We performed KEGG Pathway analysis and GO Biologic Process analysis of this cohort of genes and report here the top 10 pathways and GO Terms enriched with these differentially expressed genes (Figures 1C–F). The 30 most highly up- and downregulated of all of these genes are also shown in Table 1.

**Table 1:** Summary of RNA-seq differential gene expression in B16-F1 vs. B16-F1ΔFOXC2 melanoma.

**Top 30 DE genes upregulated in B16-F1 vs. B16-F1ΔFOXC2**							
**Gene**	**Fold change**	***p*****-value**	***q*****-value**	**Gene**	**Fold change**	***p*****-value**	***q*****-value**
*Fscn1*	20.17	1.04E-07	1.19E-05	*Iqgap*	3.65	9.60E-09	2.88E-06
*Cbr3*	9.24	3.04E-10	2.76E-07	*Ece1*	3.60	5.30E-12	6.14E-08
*Mbp*	8.53	1.71E-09	9.88E-07	*Cntln*	3.60	1.28E-11	6.14E-08
*Pdpn*	7.51	2.26E-10	2.39E-07	*Trim25*	3.59	6.35E-09	2.28E-06
*Foxr2*	7.27	1.52E-10	1.91E-07	*Zfp521*	3.54	6.63E-11	1.05E-07
*Mcoln3*	5.77	3.10E-11	7.27E-08	*Cort*	3.51	1.72E-05	0.00031
*Lmo1*	5.53	1.85E-08	4.52E-06	*Epha3*	3.45	6.81E-07	3.81E-05
*Dcdc2a*	5.10	6.71E-10	5.01E-07	*Tmlhe*	3.42	2.11E-11	6.70E-08
*Plp2*	4.88	3.24E-08	6.03E-06	*Cyp26b1*	3.39	3.12E-06	9.21E-05
*Nqo1*	4.77	3.03E-10	2.76E-07	*Pafah1b3*	3.33	6.56E-11	1.05E-07
*Taf9b*	4.67	1.58E-07	1.49E-05	*Prrg4*	3.27	1.43E-07	1.37E-05
*Nostrin*	4.64	4.77E-09	2.13E-06	*Hoxd13*	3.26	4.86E-09	2.13E-06
*Col4a1*	4.52	4.54E-08	6.95E-06	*Rnf113a2*	3.22	2.08E-07	1.77E-05
*Vgll3*	4.37	1.18E-07	1.24E-05	*Armcx1*	3.20	3.75E-08	6.45E-06
*Tor4a*	3.65	6.21E-08	8.57E-06	*Pmp22*	3.16	2.91E-08	5.68E-06
**Top 30 DE genes downregulated in B16-F1 vs. B16-F1ΔFOXC2**							
*Lgals3*	−7.73	2.22E-09	1.22E-06	*Pla2g2e*	−2.70	3.27E-05	0.00049
*Oas1a*	−6.41	9.54E-09	2.88E-06	*Ksr1*	−2.66	3.64E-09	1.78E-06
*Mgll*	−5.87	1.99E-05	0.00034	*Itgb3*	−2.66	2.85E-08	5.68E-06
*Isg15*	−4.22	3.24E-07	2.41E-05	*Pet2*	−2.63	1.45E-11	6.14E-08
*Tagln2*	−4.21	3.28E-08	6.03E-06	*Sphk1*	−2.63	2.45E-05	0.00040
*Oas1g*	−3.82	5.70E-08	8.04E-06	*Stat1*	−2.61	2.08E-07	1.77E-05
*Lcp1*	−3.59	1.76E-06	6.49E-05	*St6gal1*	−2.61	8.63E-05	0.00095
*Hsd11b1*	−3.36	1.13E-07	1.24E-05	*Met*	−2.58	0.0014	0.0074
*Mogat1*	−3.35	8.77E-07	4.51E-05	*Arfgef3*	−2.58	4.20E-06	0.00011
*Ifi27*	−3.11	1.92E-07	1.70E-05	*Tinagl1*	−2.54	5.38E-06	0.00014
*Trpm1*	−2.88	1.37E-05	0.00026	*Adam23*	−2.54	8.24E-10	5.81E-07
*Ifitm3*	−2.83	3.62E-06	0.00010	*Fam129a*	−2.54	1.50E-06	6.03E-05
*Ddx58*	−2.81	1.52E-06	6.03E-05	*Mapre3*	−2.54	7.81E-07	4.11E-05
*Xaf1*	−2.77	1.13E-05	0.00023	*Egr1*	−2.54	8.19E-05	0.00091
*Fam178b*	−2.74	6.35E-08	8.68E-06	*Ecm1*	−2.53	1.44E-05	0.00027

*List of the top 30 upregulated and downregulated genes in B16-F1 vs. B16-F1ΔFOXC2 melanoma cells as determined by RNA-seq analysis. q value = false discovery rate (FDR)-adjusted p-value*.

Our recent study highlighted several differentially expressed genes upregulated in B16-F1 that are associated with GO Terms related to the cellular response to xenobiotics and oxidative stress. We also noted in that study the downregulation in B16-F1 of several genes associated with GO Terms related to IFN responsiveness (16). Our current KEGG Pathway analysis supports these findings, with significant enrichment of B16-F1 upregulated genes in pathways related to xenobiotic metabolism and glutathione metabolism as well as significant enrichment of B16-F1 downregulated genes in pathways related to viruses and Nod-like receptor signaling, all of which include several genes involved in cellular responses to IFN. Indeed, many of the most significantly up- and downregulated genes listed in Table 1 have functions related to these particular pathways and were validated by qRT-PCR in our previous study.

Our data supports previously reported functions of FOXC2 in other cancer types and provides potential molecular insights into the oncogenic activity of this transcription factor. With regard to FOXC2's well-established role in promoting chemotherapy resistance in tumor cells (14, 15, 19, 20), our data suggests potential mechanisms by which this chemoresistance may be achieved, including induction of genes associated with drug metabolism such as the carbonyl reductase gene *Cbr3*, the oxidoreductase gene *Nqo1*, the cytochrome P450 family member *Cyp26b1*, and several members belonging to the glutathione S-transferase gene family (which fall outside of the top 30 upregulated genes shown in Table 1). Our findings are also consistent with work in other tumor and non-tumor models demonstrating a role for FOXC2 in the activation of PI3K-Akt-mTOR signaling (5, 20, 21), as we found that several genes associated with the PI3K-Akt signaling pathway (mmu04151) and the mTOR signaling pathway (mmu04150) were upregulated in B16-F1 as compared to its FOXC2-deficient counterpart. These genes include the *Pik3r2* gene, which encodes the p85β regulatory subunit of PI3K known to induce oncogenic transformation and cellular proliferation (22, 23), and the *Insr* gene, whose protein product drives various oncogenic activities through PI3K signaling (24). FOXC2 is also well-known for its ability to promote EMT and tumor cell migration/invasion (10, 25), and our findings suggest potential mechanisms by which these hallmarks of cancer progression might be regulated by FOXC2 as well. In this regard, some of the most highly upregulated genes in B16-F1 include *Fscn1* (20.17-fold upregulation) and *Pdpn* (7.51-fold upregulation). The fascin protein encoded by *Fscn1* organizes F-actin into bundles needed to form cellular protrusions that enhance tumor cell migration (26), and the actin-rich podoplanin protein encoded by *Pdpn* enhances tumor cell invasion, most likely by stabilizing invadopodia that trigger extracellular matrix (ECM) degradation (27, 28). Additionally, FOXC2-associated downregulation of genes belonging to the Focal adhesion pathway (mmu04510), such as the fibronectin-encoding *Fn1* gene and the integrin-encoding *Itgb3* gene, the latter of which is a known direct target of FOXC2 (29), may contribute to ECM remodeling and the altered adhesion of tumor cells to ECM components that occurs during the invasion process.

In addition to offering molecular insight into the previously described oncogenic activities of FOXC2, the RNA-seq dataset described herein highlights potentially novel tumor-promoting functions for this transcription factor as well. Of note, although previous work has demonstrated FOXC2-associated regulation of glycolysis (12), fatty acid oxidation (30), and mitochondrial metabolism (31), a role for FOXC2 in other metabolic pathways has not been reported to date. Interestingly, our differential gene expression analyses suggest the likelihood that FOXC2 also contributes to amino acid metabolism, as several GO Terms and Kegg Pathways related to amino acid biosynthesis and metabolism were significantly enriched with genes upregulated in the FOXC2-expressing B16-F1 cell line. Many of these genes, including *Phgdh, Psat1*, and *Psph*, play important roles in serine biosynthesis, a process that has been shown to accelerate melanoma progression and confer resistance of *BRAF* V600E mutant melanoma to the targeted inhibitor vemurafenib (32, 33). To date, only one other group has demonstrated a role for FOXC2 as a regulator of amino acid metabolism. In a recent study by Ramirez-Peña et al., FOXC2 was found to negatively regulate glutamine utilization in breast cancer cells undergoing EMT by downregulating expression of the GLS2 glutaminase (31). Our data now highlight the potential for FOXC2 to modify additional metabolic pathways in cancer cells, suggesting that this transcription factor may contribute to a variety of metabolic adaptations over the course of tumor progression.

Another previously unappreciated function of FOXC2 revealed by our data is its negative regulation of genes associated with IFN signaling, a finding that is particularly intriguing in light of recent studies demonstrating that both type I IFN and IFNγ signaling pathways within tumor cells are critical to the efficacy of cancer immunotherapies (34–37). Indeed, our recent analysis of melanoma patient TCGA data showed that *FOXC2* expression correlates negatively with progression-free survival (PFS) of patients treated with the CTLA-4 immune checkpoint inhibitor ipilimumab (16). Though the mechanism by which FOXC2 might promote resistance to checkpoint blockade therapy remains to be elucidated, it is interesting that in our murine model FOXC2 negatively regulated the expression of several IFN signaling pathway components, including the *Ddx58* gene encoding RIG-I and the *Stat1*/*Stat2*/*Stat3* and *Irf7*/*Irf9* transcription factor genes. FOXC2 expression was also associated with downregulation of various IFN-stimulated genes, including *Oas1a, Oas1g, Isg15, Ifi27, Ifi35, Ifitm3, Ifit1*, and *Ifit3*, among others. In keeping with our observation of FOXC2-associated downregulation of *Ddx58* expression and the aforementioned link between *FOXC2* expression and poor PFS of melanoma patients on ipilimumab, it is worth noting that Heidegger et al. recently demonstrated the importance of tumor cell-intrinsic activation of RIG-I in the success of checkpoint blockade therapy (38). Interestingly, RIG-I deficiency in cancer cells was also recently linked to the induction of tolerogenic dendritic cells (39), a cell type that could impact the efficacy of several immune-based therapies and one that is of particular interest to our laboratory (40, 41). We are therefore eager to explore in our model how FOXC2's negative regulation of RIG-I and other IFN pathway genes might contribute to tumor immune evasion and various forms of resistance to clinically relevant cancer immunotherapies.

It is worth noting that one potential limitation of our current study is its utilization of a murine, rather than human, melanoma cell line. Going forward, it will indeed be worth validating our findings with a similar approach in frequently studied human melanoma cell lines, such as A375 and SK-MEL-3. In order to gain additional insights into FOXC2 activity in human melanoma, we are also in the process of evaluating by immunohistochemistry how FOXC2 expression levels in melanoma patient biopsies correlate with expression of proteins of interest that have emerged from this study. Together with analyses evaluating how FOXC2 expression and subcellular localization correlate with clinicopathological features and patient outcome, these findings are likely to yield important questions related to the basic biology of FOXC2 function in melanoma that can be easily addressed in our B16-F1/B16-F1ΔFOXC2 model. Additionally, though B16-F1 is a subclone of B16 melanoma and therefore lacks the genetic diversity of a naturally arising heterogeneous tumor, it nevertheless recapitulates many features of highly aggressive human melanomas, and it has become a useful model system for investigating several hallmarks of tumor progression both *in vitro* and *in vivo* (42). Ongoing work in this model, which does not carry mutations in the *BRAF* and *PTEN* genes frequently associated with melanoma (43, 44), may be particularly relevant to understanding the progression of the still large percentage of melanomas not driven by mutations in these two genes. In this regard, that our B16-F1ΔFOXC2 model represents to our knowledge the first complete FOXC2 knockout cell line underscores the potential utility of this system for gaining important mechanistic insights into a potentially alternate driver of melanoma progression. Moreover, with evidence continuing to emerge that FOXC2 can function as an oncogenic driver of various other cancer types, comparative studies between our wild-type and complete FOXC2 knockout melanoma cell lines are likely to reveal important functions for this transcription factor that are of broad relevance to other forms of cancer as well.

In conclusion, this Data Report describes a high-quality RNA-seq dataset that we believe will serve as an important resource for investigators interested in studying the oncogenic activity of FOXC2. Importantly, our differential gene expression analyses not only offer potential molecular explanations for well-established FOXC2-driven hallmarks of cancer progression but also suggest novel tumor-promoting functions for this transcription factor. Going forward, we hope these data will invite new questions about the oncogenic functions of FOXC2 and ultimately drive future studies that aim to: (1) improve our understanding of FOXC2 activity in cancer cells and (2) inform therapeutic strategies designed to interfere with FOXC2-associated cancer progression.

## Data Availability Statement

The RNA-seq data discussed in this article have been made publicly available in NCBI's Gene Expression Omnibus under Dataset Name “RNA-seq Analysis of Differential Gene Expression in Wild-type Versus FOXC2-deficient B16-F1 Melanomas” (GEO Series accession number GSE134296).

## Author Contributions

KH was responsible for all aspects of the experimental work and writing of this article. CW contributed to the generation of the B16-F1ΔFOXC2 cell line and assisted with analysis of the RNA-seq data described herein. Both authors approved the submitted version of this manuscript.

### Conflict of Interest

The authors declare that the research was conducted in the absence of any commercial or financial relationships that could be construed as a potential conflict of interest.
